# Comparative Analysis of Time-Slotted Channel Hopping Schedule Optimization Using Priority-Based Customized Differential Evolution Algorithm in Heterogeneous IoT Networks †

**DOI:** 10.3390/s24041085

**Published:** 2024-02-07

**Authors:** Aida Vatankhah, Ramiro Liscano

**Affiliations:** Department of Electrical, Computer and Software Engineering, Ontario Tech University, 2000 Simcoe St N, Oshawa, ON L1G 0C5, Canada; rliscano@ieee.org

**Keywords:** Time-Slotted Channel Hoping, IEEE 802.15.4e, 802.15.4e TSCH scheduling, wireless sensor network, Differential Evolution (DE) optimization

## Abstract

The Time-Slotted Channel Hopping (TSCH) protocol is known for its suitability in highly reliable applications within industrial wireless sensor networks. One of the most significant challenges in TSCH is determining a schedule with a minimal slotframe size that can meet the required throughput for a heterogeneous network. We proposed a Priority-based Customized Differential Evolution (PCDE) algorithm based on the determination of a collision- and interference-free transmission graph. Our schedule can encompass sensors with different data rates in the given slotframe size. This study presents a comprehensive performance evaluation of our proposed algorithm and compares the results to the Traffic-Aware Scheduling Algorithm (TASA). Sufficient simulations were performed to evaluate different metrics such as the slotframe size, throughput, delay, time complexity, and Packet Delivery Ratio (PDR) to prove that our approach achieves a significant result compared with this method.

## 1. Introduction

Time-Slotted Channel Hopping (TSCH) is a synchronous Medium Access Control (MAC) protocol specified in the IEEE 802.15.4 standard amendment [1]. To provide more reliability to upper network layers, TSCH combines Time Division Multiple Access (TDMA) and Frequency Division Multiple Access mechanisms (FDMA) [2]. The former allows several users to share the same frequency channel by dividing the signal into different time slots, while the latter allows multiple users to send data through a single channel by dividing the bandwidth of the channel into separate nonoverlapping subchannels and allocating each subchannel to a separate user.

In TSCH, medium access is orchestrated by a schedule that is distributed to all the nodes in the network. The network coordinator is responsible for the management and control of traffic flows, and it also computes the optimized time slot and channel assignment. For each pair of nodes, a cell in the schedule is allocated to specify when and in which channel the transmissions will take place. A cell is indicated by a tuple (timeslotOffset and channelOffset), and it can be shared by multiple transmissions or dedicated to only one transmission. The standard does not define how the TSCH packet transmission schedule is defined. In [3], a thorough analysis was conducted on several existing TSCH scheduling protocols, bringing attention to their respective advantages and disadvantages.

Industrial sensor networks are expected to accommodate sensors with different packet rates, and this makes it particularly challenging to determine an optimal transmission schedule for the network. For instance, consider two sensors generating 4 and 100 packets/s, respectively. Determining an optimal slotframe length for the flows is important since a short slotframe length results in too many repetitions of a slotframe prior to the generation of any data, whereas a schedule with a long slotframe will suffer high end-to-end delays [4]. Consequently, the slotframe should be long enough to meet throughput.

The delay optimization problem is identified as a combinatorial optimization problem and NP-hard [5,6]. Due to the complexity of this optimization problem, we leveraged a Customized Differential Evolution (CDE) algorithm to determine an optimal IEEE802.15.4e TSCH schedule that minimizes delay while still meeting the required throughput for a centralized heterogeneous sensor network.

While this approach is developed for TSCH, its adaptability extends beyond this protocol. By focusing on transmissions within a single channel, it can be applied to and is applicable to any other TDMA-based communication protocols. This versatile approach has the potential to assist nodes in coordinating actions and facilitating efficient data transmissions within diverse network environments to reach minimal delay.

The Customized Differential Evolution approach was previously introduced in [7,8]; however, in this paper, we introduce an algorithm named Priority-based Customized Differential Evolution (PCDE), incorporating the following enhancements:

- We conducted extensive simulations and performed a comprehensive analysis of key metrics and direct comparisons with an alternative approach.
- We integrated a prioritization step into the CDE optimization algorithm. This step prioritizes nodes with higher packet rates or more remaining transmissions, enabling urgent data flows to be transferred first resulting in lower delays and better packet delivery.

The main contributions of this work are summarized in the following points:

- We enhanced the CDE optimization algorithm to find an optimal TSCH schedule that can meet both throughput and delay requirements. The TSCH schedule’s variable number of transmissions in each cell presents a challenge for Differential Evolution (DE) optimization. This is due to the requirement for a defined search space range with specified sizes, which is not fulfilled in this scenario.
- We generated a schedule for the networks with various traffic data rates while most other works addressed fixed packet rates. Most papers consider the fixed packet rate for sensors [9,10,11,12,13,14], and few of the existing works consider heterogeneous sensors. We optimized the throughput and minimized the delay.

This paper is structured as follows. We start with a review of related works in Section 2. Moving on, Section 3 is dedicated to explaining how we optimized slotframes by using a PCDE algorithm for TSCH. We delve into the details of the experimental setup in Section 4. The outcomes of the experiments conducted on PCDE are analyzed in Section 5. Furthermore, in Section 6, we present the simulation results and compare them to the TASA scheduler. Section 7 outlines the limitations of the proposed algorithm. Finally, Section 8 concludes this paper.

## 2. Related Works

A conflict-free TSCH scheduling algorithm was proposed in [10], which targets the minimization of transmission delays by reducing the slotframe length. The authors introduced the concept of “Wave”, which is a period where each node performs the transmission of data at least once during the Wave time. In this paper, the nodes closer to the sink suffer high traffic overflows or queue overflow, and some nodes will suffer high delays in large networks. Additionally, sensors transmit a fixed number of packets, without taking into account the heterogeneous nature of these sensors.

A debt-based scheduler is presented in [15]. In this approach, a debt value is calculated for each TSCH device that has a message to transmit, which is equal to the multiplication of the remaining number of data messages that the node has to transmit and the depth of the device in the network. No spatial reuse was applied on the cells in this paper; that is, a cell is granted to only one transmitter, although we can use spatial reuse to assign multiple transmissions in one time slot, which results in a smaller slotframe size as well as delay. Similarly, Ines Khoufi et al. [16] proposed a multislotframe to determine the lower bound number of slots required to perform data gathering and to support sensor flows with data delivery constraints. These two approaches [15,16] only allocate one cell per node to handle all traffic. Therefore, the performance of the algorithm degrades under high traffic loads.

The Orchestra scheduling approach [17], integrated into the IEEE 802.15.4 standard, provides dynamic and efficient coordination for wireless communication in LR-WPANs, enhancing resource allocation and reducing interference. However, its advantages must be weighed against its limitations, particularly when dealing with a high number of nodes. In such scenarios, Orchestra scheduling may introduce complexities and delays, impacting network performance and introducing coordination challenges.

The scheduler named the Optimized Scheduling Cell Allocation Algorithm for convergecast (OSCAR) in [18] leverages Orchestra principles. This scheduler is designed with the assumption that nodes closer to the root experience higher traffic loads during convergecast. Nodes are assigned a class based on their rank, with those closer to the root receiving extra cells. Allocated cells decrease with a reduced traffic load but are reallocated if the traffic load increases again. Consequently, OSCAR may allocate unnecessary cells in unbalanced network scenarios. Additionally, this approach considers fixed packet rates.

In [19], the objective is to enhance Orchestra’s receiver-based scheduling policy by adjusting the static schedule to accommodate high traffic loads or bursts. This methodology primarily targets the immediate child nodes connected to the gateway, responsible for transmitting network traffic. When a child node’s buffer exceeds a specified threshold, it notifies the gateway of potential congestion. In response, the gateway increases reception time slots for that specific child node by using a hash function. The quantity of additional allocated time slots is proportionate to the size of the subtree rooted at the child node. However, this approach may introduce scalability issues as it addresses collision concerns solely at the gateway, leaving the problem unresolved in the other nodes throughout the network.

A centralized Adaptive Multi-hop Scheduling (AMUS) algorithm was proposed in [20] to provide optimized schedules using tentative cell allocations. AMUS reserves additional cells for those links that might be heavily loaded or prone to interference to improve communication reliability and achieve low latency.

In [21], a distributed TSCH scheduler was implemented. This scheduler designates a shared slot for synchronization and new node connections, while separate slots are randomly allocated for communication. If interference disrupts packet transmission, a node switches from sending to listening mode, enabling the scheduling of a new connection. However, this transition consumes both time and energy since it necessitates the radios to constantly either receive or transmit.

The Traffic-Aware Scheduling Algorithm (TASA) [22] aims to find the minimal number of slots needed to send all data to a root node. To reach this goal, matching and coloring functions are used to plan the distribution of slots and channels across the entire network without any collision. First, links that still have data to transmit are selected at the corresponding time slot through a matching process. Then, the channel offsets of the links are allocated so that interference does not occur through the matching process. We compared our algorithm against TASA. TASA creates a collision-free and interference-free schedule by using graphs or metaheuristic algorithms aiming to minimize energy consumption. The TASA algorithm can be relatively complex, especially when dealing with high packet ratios. To handle this complexity, we utilize the PCDE optimization algorithm, which guarantees the creation of a collision- and interference-free schedule while focusing on minimizing both the delay and expected throughput. Moreover, TASA does not provide any guarantees in terms of reliability because it assumes perfect links and no retransmissions. Consequently, it is possible that the required throughput is not met due to several reasons such as packet loss, etc. To address this, we incorporate the concept of a reserved slot for each node within a slotframe to account for the probability of packet loss. Moreover, we used a cosimulation of Matlab and TSCH-SIM to simulate the network in a real environment and make sure the expected throughput was met.

A Combinatorial Multiarmed Bandit (CMAB) was proposed in [23], which determines the optimal scheduling by assignment of TSCH cells to links using the Linear Learning Rewards (LLR) algorithm. This is performed by using a bipartite graph that matches noninterfering links to slotframe matrix cells. We also used a graph to represent the noninterfering links, but we optimized this by using a DE algorithm.

The authors propose Adaptive Static Scheduling in [24] to allow each pair of nodes to control their active time slots to improve the energy efficiency of the TSCH networks in a distributed manner. A static scheduler is built at compilation time with excessive time slots over allocation, yet the nodes can dynamically activate or deactivate their a priori allocated time slots according to the traffic requirements. However, the authors did not consider the cost of blind overprovisioning in terms of the delay performance.

## 3. Priority-Based Customized DE Optimization

In this section, we provide an overview of the Priority-based Customized DE optimization algorithm. Derived from the traditional Differential Evolution algorithm [25], we outlined the modifications to enhance its effectiveness. For a comprehensive understanding of this approach, detailed information about optimization phases can be found in [7].

In this paper, prioritization was integrated into CDE based on the node’s pending packets and packet rate. We calculate this value for participating nodes in a time slot to achieve several advantages, including facilitating a quicker termination of the optimization process. Instead of relying on the random generation of candidate schedules during optimization, prioritization offers an intelligent selection of transmissions, leading to a shorter time needed to meet termination criteria. The transmission of nodes with high packet rates is more challenging to assign within the schedule due to the need for more frequent repetitions. By prioritizing these nodes first and adding other transmissions accordingly, the optimization iterations required until the criteria are met are reduced. Conversely, without prioritization, assigning a low-priority transmission may prevent the assignment of another high-priority transmission, necessitating an increase in the slotframe to accommodate the remaining transmissions. Therefore, it can be concluded that prioritization not only facilitates a quicker termination of the optimization process but also contributes to reducing the slotframe size.

**Figure 1:**
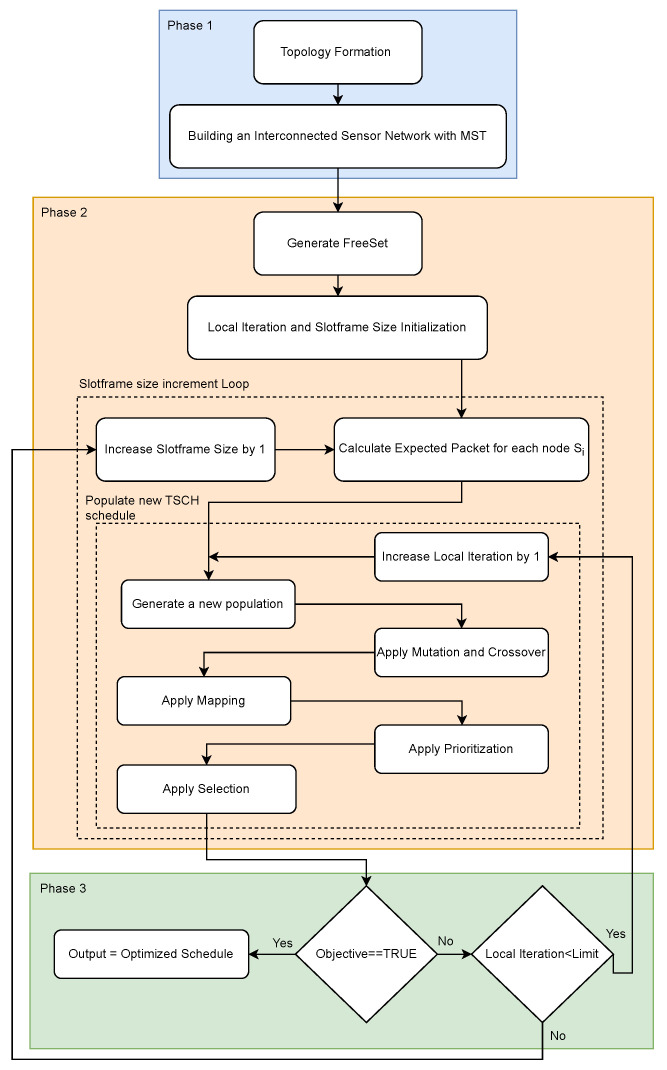
Priority-based Customized Differential Evolution Algorithm flowchart.

Figure 1 illustrates the general flowchart of the PCDE algorithm. In this flowchart, Phase 1 initiates with the random deployment of nodes forming the network topology. Leveraging the Minimum Spanning Tree (MST) concept, a connected tree structure is established. Phase 2 focuses on the generation of collision- and interference-free sets and the subsequent creation of a new TSCH schedule. In Phase 3, we evaluate both the termination criteria and the Local Iteration loop. If the termination criteria, which involve meeting an expected throughput target, are satisfied, the necessary output files are generated. These phases are elaborated further in the following sections.

### 3.1. Topology Formation and Building an Interconnected Sensor Network with MST

The algorithm persists in generating a random topology until it achieves a connected sensor network, where all sensor nodes are intricately interconnected, facilitating seamless communication and information exchange between any node pair [26]. The Minimum Spanning Tree approach is employed to interconnect all nodes in the network, ensuring a cycle-free structure. In a sensor network, cycles in the network topology can lead to redundant data transmission and processing. Using an MST cycle-free structure avoids such redundancy. Additionally, in order to determine the suitability of assigning transmissions within a single time slot or an identical cell within the TSCH schedule, we must establish a connected network that defines the routing paths. The Minimum Spanning Tree offers an efficient solution to guarantee the connectivity of all nodes in the network.

Algorithm 1 outlines the procedure for generating a connected MST. The algorithm’s primary objective is to create a topology for *N* nodes until a connected MST is established. As shown in Algorithm 1, nodes are placed within a 2D area, leading to the computation of the adjacency matrix Dist. Using Dist, the algorithm forms the mesh graph *G*, interconnecting all node pairs. For each pair of sensor nodes *i* and *j*, Dist(i,j) calculates the Euclidean Distance between node $S_{i}$ and $S_{j}$. If this distance exceeds the specified sensing range *R*, a value of 1000 signifies that communication is not possible. The variable Flag serves as an indicator of tree connectivity and starts at 0. As the algorithm progresses and identifies a connected tree, Flag is updated to one, signaling the successful completion. The Minimum Spanning Tree *T* is generated from *G*, with edge weights T.Edge between nodes *i* and *j* set to corresponding Dist(i,j) values. An edge weight of 1000 in the tree indicates nonconnectivity, highlighting node pairs situated beyond the sensing range. If all edge weights within the generated MST *T* are in the sensing range *R*, Flag is set to one, indicating the successful establishment of a connected populated tree *T*.

**Figure 2:**
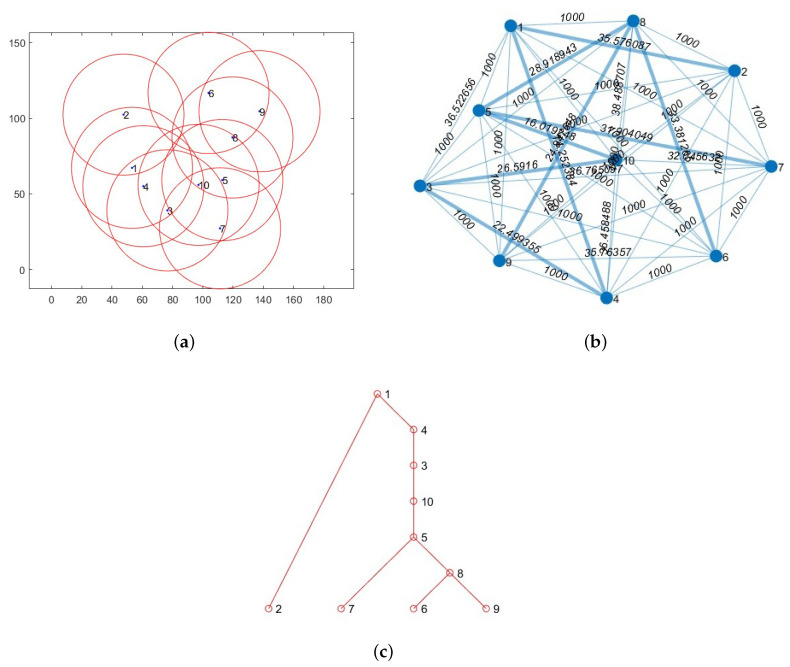
(**a**) Randomly generated topology with 10 nodes. (**b**) Designated feasible routes for Spanning Tree in Mesh graph. (**c**) Spanning Tree for 10 nodes.

Figure 2a exhibits an instance of a randomly generated topology featuring ten nodes. With a sensing range set to 40 m, the nodes in the mesh graph, as shown in Figure 2b, positioned farther than 40 m represent locations beyond the communication range. These particular edges are allocated a path cost of 1000. Notably, the edges that link the nodes within the reachable range are distinctly highlighted in bold blue within the same figure, as shown in Figure 2b. As a result, the resulting Spanning Tree is presented in Figure 2c.

### 3.2. Generating TSCH Schedule Using Priority-Based Customized DE Optimization for Heterogeneous Networks

**Figure 3:**
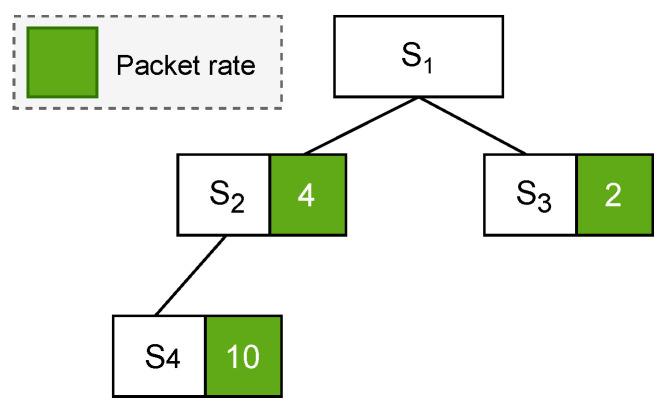
Tree topology example with root and three sensor nodes.

To illustrate the operational principles of the PCDE algorithm, a simple tree topology is depicted in Figure 3. This topology comprises a root node denoted as $S_{1}$ and three additional sensor nodes labeled as $S_{2}$, $S_{3}$, and $S_{4}$. The green box associated with each sensor node in Figure 3 represents the node’s packet rate, indicating the number of packets that the node generates in one second.
**Algorithm 1** Connected Spanning Tree Generation  1:Flag←0  2:NotInRange←1000  3:**while** Flag==0 **do**  4:   Distribute *N* nodes in a 2D area of 200 ∗ 200 m^2^  5:   **for** i=1 to *N* **do**  6:     **for** j=i+1 to *N* **do**  7:        $Dist\left(i,j\right)\leftarrow EuclideanDistance\left(S_{i},S_{j}\right)$  8:        **if** Dist(i,j)>R **then**  9:          Dist(i,j)←NotInRange10:        **end if**11:     **end for**12:   **end for**13:   G← Generate Graph *G* using matrix Dist14:   T← Generate MST Tree *T* using Graph *G*15:   **for** each T.Edge between $S_{i}$ and $S_{j}$ **do**16:     T.Edge(i,j).Weight←Dist(i,j)17:   **end for**18:   **if** All(T.Edge.Weight)≠NotInRange **then**19:     Flag←120:   **end if**21:**end while**22:**return** *T*

#### 3.2.1. Create Collision- and Interference-Free Sets

**Figure 4:**
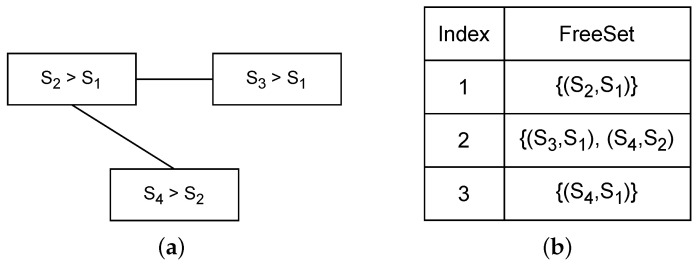
(**a**) Union of collision and interference graph. (**b**) FreeSet.

The collision and interference graphs are defined based on the routing table and the neighborhood of sensor nodes. By taking the union of the collision and interference graphs, as depicted in Figure 4a, the FreeSet is determined, representing all sets of transmissions that can be assigned to the same time slot or a single cell in the TSCH schedule. FreeSet is demonstrated in Figure 4b.

#### 3.2.2. Populate New TSCH Schedule

In this phase, we leverage the PCDE algorithm to create a schedule for a centralized heterogeneous sensor network. This schedule is specifically designed to meet the required throughput in a network with different packet rates, aiming to minimize delay. Through each iteration of the optimization process, we accurately estimate the number of packets expected to be generated within the temporary chosen slotframe size. Then, the best schedule in terms of the highest possible throughput is built by using the chosen slotframe size. At the end of each optimization iteration, we construct a schedule that maximizes the inclusion of the required transmissions with no collision and interference in the chosen slotframe size.

The enhancement we introduced to the CDE optimization involves the integration of a prioritization step within the selection phase of optimization before evaluating the schedule’s fitness value. The assignment of sets from the FreeSet to cells within the TSCH schedule guarantees the prevention of collisions or interference between transmission pairs within the cell. While the concern for interference between cells in the same time slot is mitigated by channel hopping, the potential persists for collisions between transmissions originating from a specific cell and those arising from other cells sharing the same time slot. Considering two transmissions, $S_{i}\to S_{j}$ and $S_{k}\to S_{p}$, both take place within the same time slot but on separate channel offsets. Collisions can arise under two scenarios:

- When a node is scheduled to send and receive at the same time slot. For instance, if i=p or j=k, a collision will occur.
- When two nodes simultaneously transmit packets to the same recipient. For instance, if j=p, this condition is met, resulting in a collision.

In either situation, we possess the flexibility to choose one transmission from the colliding pair based on a priority evaluation. The priority of the transmission $S_{i}\to S_{j}$ in time slot *t* is computed by using the equation

(1) $$P_{t}\left(S_{i}\to S_{j}\right)=\sum_{ts=1}^{t}\left(EP\left(S_{i}\right)-NumAssigned\left(S_{i}\right)\right)$$

Here, $EP(S_{i})$ represents the projected number of packets from node $S_{i}$, and it is calculated by using the packet rate value (detailed in [8]). Furthermore, $NumAssigned(S_{i})$ indicates the overall count of cells assigned to node $S_{i}$.

The transmission with the higher priority is chosen while the other transmission is omitted from the schedule’s time slots. This approach ensures that transmissions with elevated priority are consistently retained. This strategic choice of prioritization accelerates the attainment of the Customized DE optimization’s termination criteria, which is set to attain the required throughput.

### 3.3. Termination Criteria

The optimization process terminates when the generated schedule successfully encompasses all the required transmissions and reaches the required throughput. If this condition is not met, the slotframe size is increased by one, and a new attempt is made to populate the schedule with the expanded slotframe size. This iterative approach allows for dynamic adjustments, ensuring comprehensive coverage of all the necessary transmissions within the schedule.

## 4. Experimental Setup

### Evaluation Approach

Given the heterogeneous nature of wireless sensor networks, the packet rate (PR) of the sensor nodes varied, resulting in varying levels of traffic. Scenarios involved sensors with low to high packet rates, leading to increased traffic volume. In this paper, we considered three different ranges for the packet rates: [0.1 0.5], [0.5 1], and [1 5], which are called low (L), medium (M), and high (H) traffic, respectively, in this paper.

Networks with sizes of 10 to 50 nodes were populated and distributed within a 2D square area of 200×200 m^2^. Each node was placed in a random position in this environment with a fixed sensing range of R=40 m.

The number of neighbors for each node varied, and to represent the overall average number of neighbors across all sensors, we employed the notation ($AVG_{NBR}$). Considering that the schedule is built according to the network topology, we analyzed the performance of the two algorithms in several scenarios that are shown in Table 1.

We assumed a standard duration of 10 ms for each time slot, and the number of available channel offsets ($n_{ch}$) was set to 4. A summary of the evaluation parameters and their specified ranges is shown in Table 2.

After populating 360 random network topologies considering the ranges specified in Table 2, we grouped them into 18 scenarios presented in Table 1. The primary objective across all scenarios is to transmit all the generated packets to the root node successfully with a minimum slotframe size. We conducted several experiments, and for each experiment, a tree topology with different densities of sensor nodes, tree depths, and packet rates was employed.

The implementation of both the TASA and PCDE approaches was performed by using Matlab. To assess the performance of these algorithms and evaluate their effectiveness based on several metrics, we utilized TSCH-SIM [27]. TSCH-SIM enables the real-time simulation of networks and provides an in-depth analysis of the network performance.

TSCH-SIM is a network simulator capable of accommodating TSCH technology. However, to further enhance its capabilities, significant modifications were essential to enable the manual specification of TSCH schedules and static routes, an aspect that was absent in its original version. The details of these enhancements can be found in [28].

**Figure 5:**
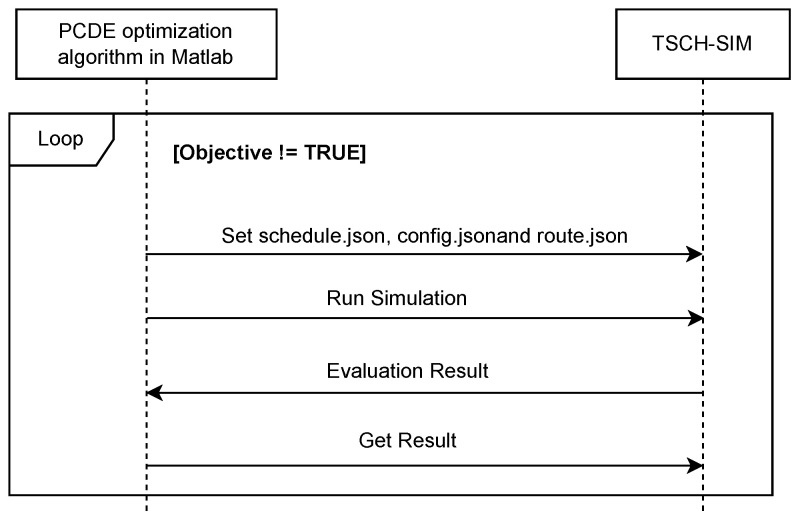
Cosimulation: sequence diagram of PCDE optimization algorithm and TSCH-SIM.

As depicted in Figure 5, our implementation leveraged Matlab to create the required JSON files for the execution of TSCH-SIM, namely “config.json”, “schedule.json”, and “routes.json”. These three files serve as the input for TSCH-SIM during the simulation process. “config.json” contains comprehensive network configuration details, including sensor node positions, PR values, connections, etc. The optimized schedule, encompassing time slot and channel offset values for each transmission, is stored in “schedule.json”. Lastly, “routes.json” specifies the designated routes within the network.

In TSCH-SIM, we utilized the generated files derived from the Matlab simulations as inputs to establish the network parameters. Through the “config.json”, “schedule.json”, and “routes.json” files, we defined the nodes’ positions, connections, schedules, and routes. In our radio propagation modeling, we selected the Logistic Loss model. This choice reflects our specific emphasis on assessing the schedule’s efficacy in reducing delays and attaining target throughput, rather than on how reliable it is to adapt to data loss. All parameters, including the time slot duration, slotframe size, sensing range, and others, which were employed in Matlab, were precisely replicated within TSCH-SIM to ensure consistent environmental conditions. Upon completion of the simulation, the evaluation results are transmitted to Matlab. If the achieved throughput matches or exceeds the expected target throughput, the algorithm terminates. However, if the achieved throughput falls short, the algorithm will persist until it discovers the optimal schedule.

Table 3 illustrates the simulation parameters and values we considered for the simulation. In Table 3, APP_WARMUP_PERIOD_SECOND is the time period it takes for all the sensor nodes to join the network (i.e., when the network is stable). Data packets are not generated before this warmup period has expired, resulting in more accurate metrics. The MAC retransmissions were left as the default value of 7, and the MAC queue size was set to 20.

## 5. Priority-Based Customized DE Optimization Algorithm Analysis

### 5.1. Fluctuation in Number of Satisfied Nodes

Throughout multiple experiments during the PCDE optimization process, we observed fluctuations in the number of nodes categorized as “SATISFIED”. Nodes classified as “SATISFIED” refer to those that have been assigned to the schedule in a quantity equal to or greater than the required number of assignments according to their packet rate.

**Figure 6:**
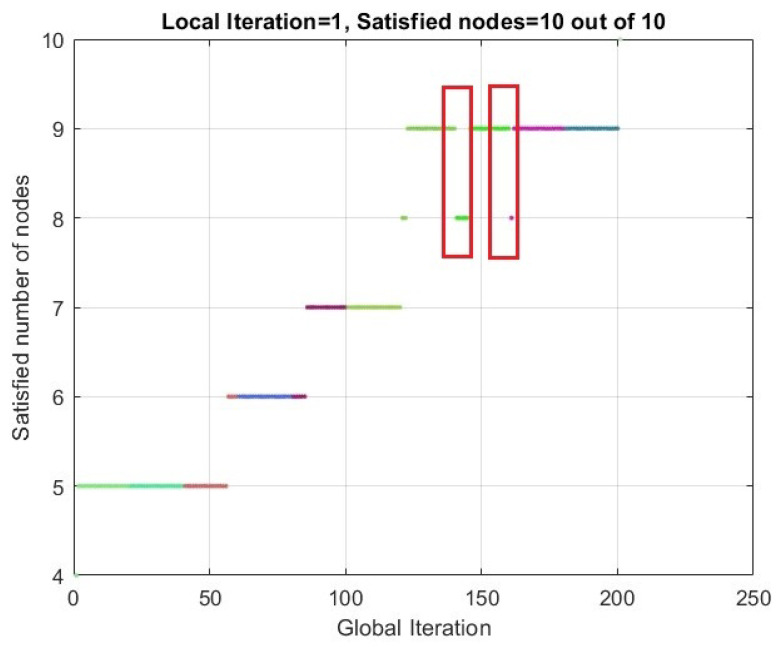
Trend of number of SATISFIED nodes during PCDE optimization process for 10 nodes.

As depicted in Figure 6, we employed different colors to highlight the optimization iterations associated with a specific slotframe size. Within this figure, it can be observed that the initial count of “SATISFIED” nodes was five. However, despite 20 Local Iterations of attempting to find a schedule with the specified slotframe size, no schedule was found to fulfill the objective of satisfying all nodes. Consequently, the slotframe size was increased by one in order to continue the optimization process. In such a scenario, when the slotframe size is increased, it is expected that the number of “SATISFIED” nodes may decrease. In the optimization trend that is shown in Figure 6, this occurred twice, which is shown as a red box. This is because the increase in the slotframe size can result in an elevation of the expected packet count for certain nodes. Consequently, it may also raise the expected packet count for their parent nodes, as the parents are responsible for relaying the packets received from their child nodes. This increment in expected packets may continue until reaching the root node. In such cases, the count of SATISFIED nodes may significantly decrease initially. However, over a few iterations with the new slotframe size, this number tends to increase again.

### 5.2. Overscheduling and Robustness

Overscheduling in PCDE optimization occurs because it encompasses high-priority pairs and attempts to accommodate as many transmissions as possible within each time slot without causing collisions or interference. This section aims to demonstrate the positive impact of overscheduling. While the packet rate of the sensor nodes is defined during the network’s initialization phase, the exact timing of packet generation remains uncertain. Consequently, there is a high likelihood of nodes failing to transmit their packets within the scheduled time slots.

The schedule generated by the PCDE optimization algorithm includes additional transmission assignments known as reserved cells. Sensor nodes can encounter various reasons for packet transmission failures. For instance, a node may fail to transmit when the packet has not yet been generated. The worst-case scenario occurs when a node is scheduled right before its packet-generation time. Consider a slot length of 10 ms and a slotframe length of 70 ms. If a node generates a packet every 60 ms and it is scheduled to send a packet only in the fifth time slot, it will miss the scheduled time slot as the packet has not yet been generated. Consequently, the node must wait for the next scheduled time slot, resulting in a delay of 70 ms. However, with an additional scheduled time slot, the delay would significantly decrease.

### 5.3. Local Iteration Value

A crucial metric in PCDE optimization is the “Local Iteration” value (LI), which signifies the maximum number of iterations that the algorithm undergoes to generate a schedule that achieves the specified objective. While increasing the Local Iteration value may lead to higher time complexity, it also presents a greater likelihood of discovering optimal schedules in lower slotframe sizes since a higher value for LI means generating more candidates for the schedule; therefore, the chance of identifying a suitable schedule before transitioning to a new slotframe size arises. It is important to highlight that expanding the slotframe size can lead to a higher number of expected packets for each node, introducing greater complexity in finding the optimal schedule.

For our analysis, we set Local Iteration values of 20 and 50 for each scenario to assess the impact of the Local Iteration value on both the time complexity and slotframe size.

**Figure 7:**
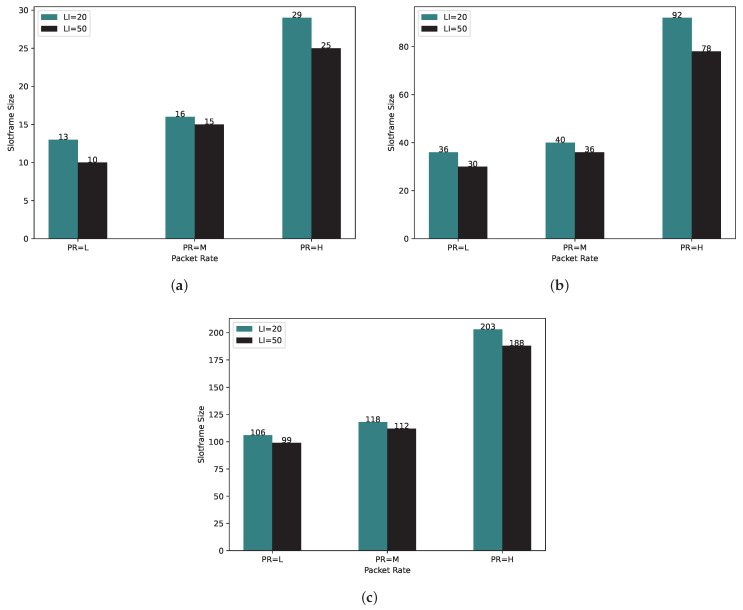
Slotframe size for Local Iteration = 20 and 50 with (**a**) 10, (**b**) 20, and (**c**) 50 nodes.

To gain insights into the slotframe size’s behavior, we conducted PCDE algorithm runs with LI values of 20 and 50 in different scenarios. As illustrated in Figure 7, the slotframe size exhibits a lower value when LI = 50 compared to LI = 20. This observation can be attributed to the higher probability of finding an optimal schedule when the schedule undergoes mutation, crossover, and selection processes, resulting in a greater chance of identifying a candidate for the optimal schedule. As depicted in Figure 7a–c, the variance in the slotframe size between LI = 20 and LI = 50 is notably more pronounced, especially when considering PR = H.

## 6. Experiments Results

In this section, we begin by introducing the evaluation metrics employed in our experimental analysis. Additionally, we present the outcomes of our experiments for PCDE, utilizing Matlab and TSCH-SIM. As detailed in Section 4, we generated and examined 360 diverse network topologies, organizing them into 18 scenarios, as shown in Table 1, to clarify the results. Our analysis encompassed several assessment metrics, each of which are concisely outlined in the subsequent discussion.

Furthermore, a comprehensive comparison of multiple metrics was conducted between PCDE and TASA to evaluate the performance efficiency of each algorithm. The overall performance results captured from these two algorithms in various scenarios are illustrated in Table 4 and Table 5. A further detailed comparison analysis is performed following the table in Section 6.1, Section 6.2, Section 6.3, Section 6.4, Section 6.5 and Section 6.6. The subsequent definitions provide an explanation of the metrics considered in our evaluation.

- Delay: Network delay refers to the total time (propagation, transmission, queuing, and processing period) a packet takes to travel from a source node to a destination node, and it is estimated in seconds. The delay is evaluated by taking the difference between the time a packet is generated and is successfully received by the root node. The average delay is calculated by utilizing Equation (2);
  (2) $$Delay=\frac{\sum_{i=1}^{N}\left(time{\left(i\right)}_{received}-time{\left(i\right)}_{generated}\right)}{total\;packets}$$
- Reliability: reliability relates to the network’s ability to transfer data successfully between the sender and receiver, and it is typically measured by using an end-to-end Packet Delivery Ratio (PDR).
- Throughput: throughput is influenced by the payload size, and it is calculated by the amount of data received successfully in a given time period, which is presented in the following formula:
  (3) $$Overall\;throughput=\frac{\sum_{i=1}^{N}received\;packets_{i}}{total\;simulation\;time}$$
  where *N* denotes the total number of packets and $received\;packets_{i}$ is the number of packets received by sensor $S_{i}$.
- Time complexity: time complexity refers to the computational efficiency of an algorithm, specifically the amount of time it takes to execute and produce a solution that satisfies the specified requirements.
- Duty cycle: this metric is defined by calculating the ratio between the length of the schedule and the slotframe size.
- Slotframe size: the size of the slotframe plays a crucial role in determining the delay, and it is determined by the total number of time slots contained within the slotframe.

### 6.1. Experiment 1: Delay

To show the effectiveness of the algorithm, we measured the average delay. This value is affected by the number of nodes, tree depth, packet rate, and the average number of neighbors significantly. Considering the same number of nodes and packet rate for two random topologies, the tree topology with a larger depth value or the higher average number of neighbors will have a higher delay since the packets should wait longer to reach the root node, and it has to pass through more hoops. Additionally, a larger value for the average number of neighbors causes a higher chance of collision and interference, and a lower number of nodes can be encompassed in one time slot.

**Figure 8:**
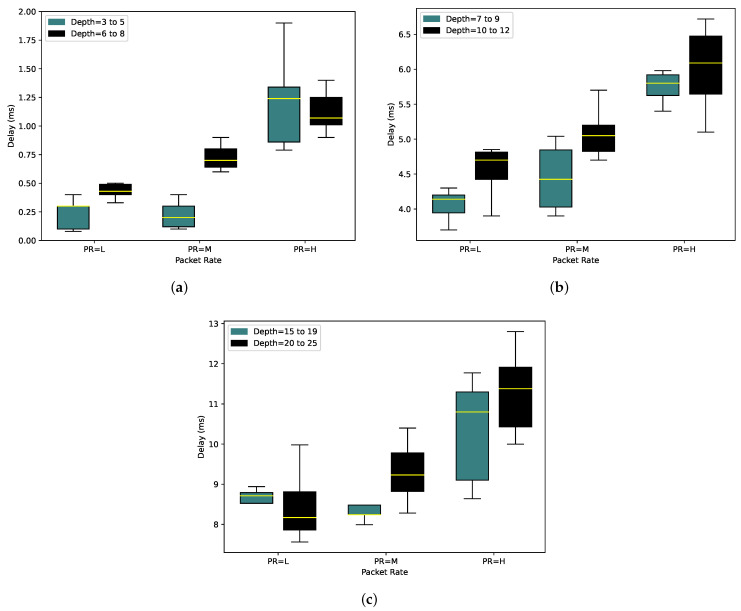
Delay for different tree depths and packet rates in networks with (**b**) 10, (**b**) 20, and (**c**) 50 nodes.

Figure 8 illustrates the delay for 10, 20, and 50 nodes. The Y axis represents the delay values while the X axis categorizes the packet rates into three groups of low (L), medium (M), and high (H). Each packet rate group is represented by two boxes. The blue boxes represent slotframe sizes for trees with shallower depth while the black boxes depict slotframe sizes for trees with greater depth.

As illustrated in Figure 8, there is a noticeable increase in the delay as the tree depth increases. This is primarily due to the fact that packets need to pass through more hoops, resulting in prolonged waiting times for delivery to the destination. Additionally, the slotframe size for such scenarios is larger, a topic we will discuss further in subsequent experiments.

For the specific case of 10 nodes illustrated in Figure 8a, it is notable that the mean delay is 0.23 ms, with almost half of the tests producing values lower than this benchmark when the depth is less. As the depth increases, this mean delay value climbs by 0.2 ms. Comparing different packet rates, in scenarios with lower tree depth, PR = M and PR = L exhibit nearly identical delay values. However, in cases with higher tree depth, the delay increment for PR = M is 0.7 ms. For PR = H, 75% of the tests result in delays of 1.44 ms or less in lower-depth scenarios. Interestingly, this value is quite close to the results observed in higher-depth scenarios, where 75% of the tests yield a delay of 1.25 ms. Remarkably, PR = H encompasses tests with random packet rates between 1 and 10 packets per second. It is probable that only high packet rates be chosen for a test, resulting in higher delays, which could affect the mean value. Consequently, the mean value for higher depth scenarios is lower than that for depth = 3 to 5. However, it is important to emphasize that even in cases of higher depth, the minimum delay observed in the tests remains elevated. Likewise, in Figure 8b,c, it becomes evident that as both the depth and packet rate increase, the delay also experiences an increase.

**Figure 9:**
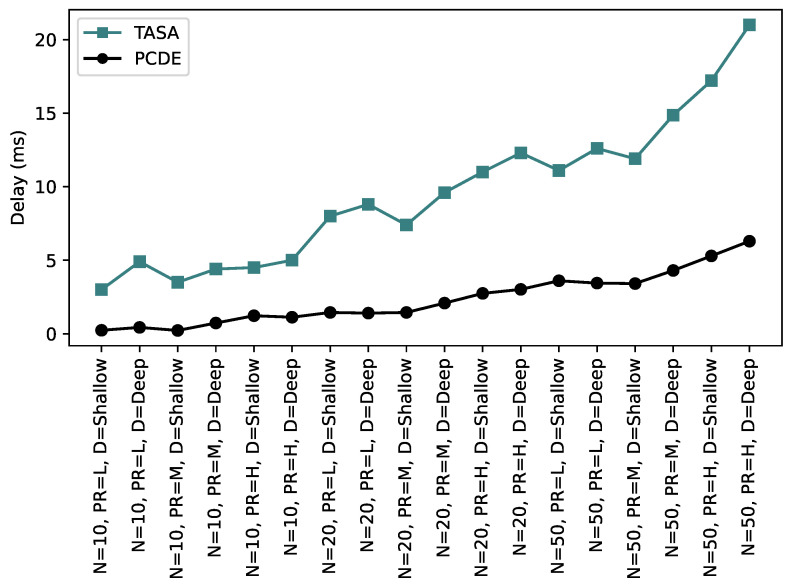
Delay comparison between PCDE and TASA.

In Figure 9, a comparison is made between the delay values of two approaches: PCDE and TASA. The figure clearly illustrates that the delay values for TASA consistently exceed those of PCDE. This discrepancy can be attributed to the fact that TASA does not consider packet delivery time, similar to our PCDE approach, where it is assumed that packets are generated during network initialization. As a result, real-time queue calculations are not performed in TASA, with the assumption that all packets are readily available for transmission or relaying. If a packet has not been received yet, it cannot be relayed to other nodes, leading to prolonged waiting times. In contrast, our PCDE approach leverages reserved time slots, increasing the likelihood of compensating for missed slots. Due to a higher probability of finding a scheduled slot for transmission in PCDE, the delay value is significantly lower compared to TASA.

### 6.2. Experiment 2: Reliability

The Packet Delivery Ratio is a critical parameter as it indicates the successful arrival of packets at the destination. Certain applications demand high PDR values; thus, we measure this parameter to assess the network’s reliability across various packet rates and sensor node densities. This allows us to measure the network’s performance and suitability for different application requirements.

**Figure 10:**
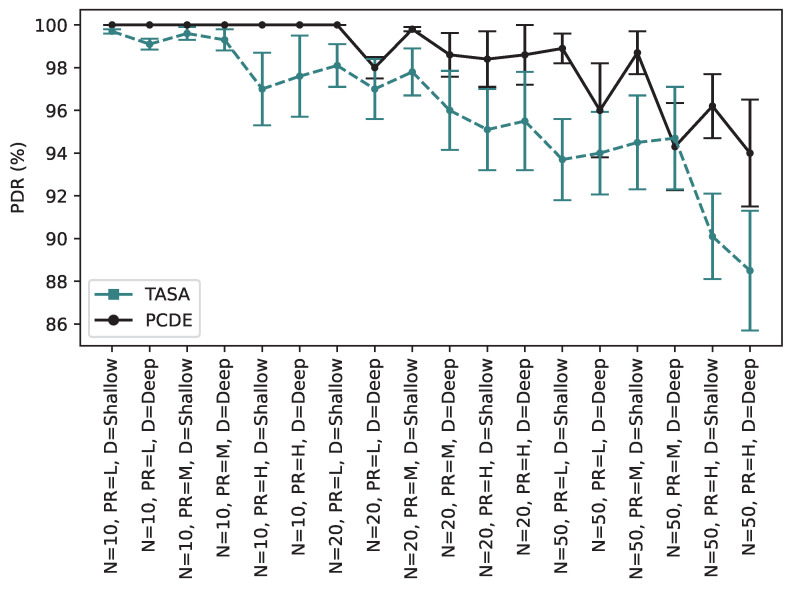
Packet Delivery Ratio comparison between PCDE and TASA.

As presented in Figure 10, the PDR value for PCDE remains consistently at 100% for all packet rates when dealing with 10 nodes. However, this figure exhibits minor fluctuations ranging between 100% and 98.4% for scenarios involving 20 nodes. Notably, the PDR experiences a more significant decline when scaling up to 50 nodes. Nevertheless, when comparing these PDR values to those detailed in Table 5, it becomes evident that our approach outperforms the alternatives.

As mentioned earlier, prioritization allows for the scheduling of nodes with a high packet rate or a high number of pending packets first. Given that these nodes are more likely to cause queue overflow and consequent packet loss due to their higher packet-generation rate, addressing them first enhances the overall Packet Delivery Ratio as well; however, this prioritization strategy ensures that nodes with lower priority are not neglected. Any available space that cannot be filled with high-priority nodes is assigned to lower-priority nodes.

In TASA, the number of transmissions aligns with the required number of transmissions for each node. Considering that a packet must arrive and be ready for relay by upper nodes within a scheduled time slot, if a packet’s transmission is scheduled, but the packet has not been received in time, the transmitting node cannot send it. It is conceivable that after missing the scheduled time slot, the node may wait for an extended period to find another available time slot for transmission.

Assuming a slotframe size of SF, if a node fails to transmit in time slot 1, it needs to wait for (SF−1)×10 ms (assuming a time slot size of 10 ms) before attempting transmission again. During this waiting period, the packet remains in the queue. Notably, the queue has limited capacity, and packets have a restricted time window for queue retention. Consequently, if a packet remains in the queue for an extended duration, it may eventually be dropped. This, in turn, has a negative impact on the PDR value.

In contrast, our approach leverages reserved slots, guaranteeing at least one extra transmission opportunity within each slotframe. Consequently, any missed packet can be promptly transmitted during the current slotframe, eliminating the need for prolonged waiting periods until the next slotframe.

As shown in Figure 10, comparing the PDR values for PCDE and TASA, it can be stated that by increasing the number of nodes, the PDR value has a relatively downward trend. Our approach improves PDR by applying priority and overscheduling transmissions, although it can be observed from this figure that in scenarios with deep depth, the PDR still drops slightly. This decrease is more notable in TASA. The PDR is affected more especially when either the network depth is deep or the packet rate is high or both of these specifications are included in a scenario simultaneously, which causes a decrease in the PDR value.

As depicted in Figure 10, when comparing the PCDE and TASA approaches in terms of PDR, it is evident that an increase in the number of nodes leads to a relatively downward trend in PDR values. Despite our approach to enhancing PDR through the application of priority and overscheduling transmissions, the figure reveals a slight decline, particularly in scenarios with a deep network depth. This decrease is more pronounced in TASA. Notably, the PDR is more affected when the network depth is deep, the packet rate is high, or both of these specifications are present concurrently in a scenario, resulting in a reduction in the PDR values.

### 6.3. Experiment 3: Throughput

**Figure 11:**
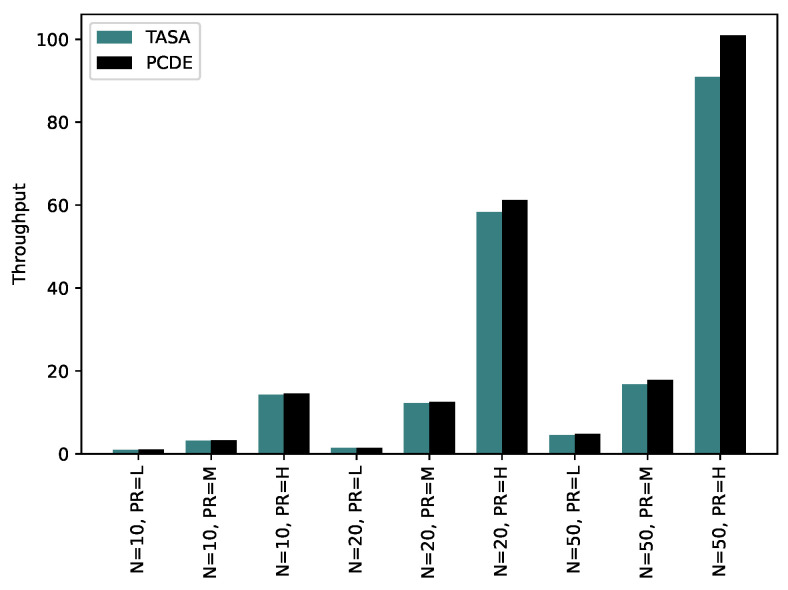
Throughput comparison between PCDE and TASA.

Throughput relies on the number of packets received during the simulation period. Therefore, the number of packets generated plays a significant role, typically increasing with the packet rate and the number of nodes, as shown in Figure 11. When comparing the throughput between the two approaches, PCDE and TASA, it becomes evident that PCDE achieves a higher throughput. This can be attributed to the fact that PCDE assigns more transmissions, resulting in higher throughput values. In contrast, TASA allocates transmissions based on the queue value, determined during the initialization step. For instance, if a node is scheduled in time slot 1 to relay a packet in TASA, it must wait in the queue list because the packet has not been received yet. This waiting time introduces the possibility of packet drops. As a result, a reduced number of received packets will lead to decreased throughput values.

### 6.4. Experiment 4: Time Complexity

**Figure 12:**
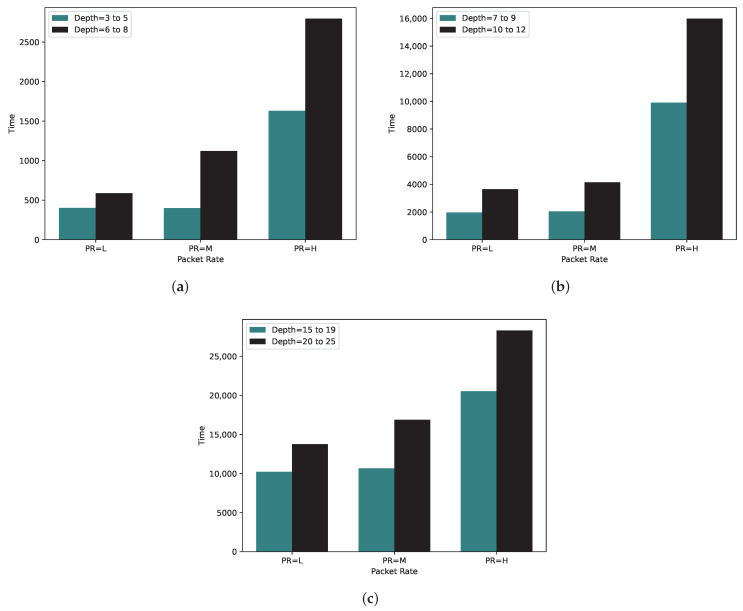
Average time complexity for (**a**) 10, (**b**) 20, and (**c**) 50 nodes in various packet rates.

We conducted the time complexity experiment to analyze three approaches in terms of the time required to find a schedule that can meet all the nodes’ throughput. The analysis of time complexity for 10, 20, and 50 nodes across various depth groups is depicted in Figure 12a–c, respectively. It can be stated that increasing either the number of nodes or the depth of the tree significantly affects the time required to find a solution for the TSCH schedule.

Providing that there was a change in the network topology or modification in QoS, the applied schedule will not work. Consequently, a new schedule must be generated according to the new conditions. The result of this experiment demonstrates how much time is required for the new schedule to be populated. Although some packets will be missed in the case of any change in the network, the algorithm that can populate a new schedule quicker according to the updated network requirement would be more efficient.

### 6.5. Experiment 5: Duty Cycle

The duty cycle is computed as the ratio between the length of the schedule and the slotframe size. According to [22], a fixed slotframe size is considered for TASA as 720 while the time slot duration is 4 ms. To have a consistent environment to evaluate the performance of these two algorithms in a fair condition, we kept the slotframe size the same at 720, while the time slot duration was set to 10 ms. Since our goal is to find a schedule with the minimum slotframe size, the found slotframe size will be equal to the schedule size. In this condition, the duty cycle for the PCDE optimization approach is always 100%.

The duty cycle is calculated as the ratio between the schedule’s length and the slotframe size. In TASA [22], a fixed slotframe size of 720 is adopted, along with a time slot duration of 4ms. For a fair performance evaluation of these two algorithms within a consistent setting, we maintained the slotframe size at 720 while adjusting the time slot duration to 10ms. Given our aim to discover a schedule with the smallest slotframe size, the determined slotframe size aligns with the schedule size itself. Consequently, the duty cycle for the PCDE approach is consistently 100%.

### 6.6. Experiment 6: Slotframe Size

**Figure 13:**
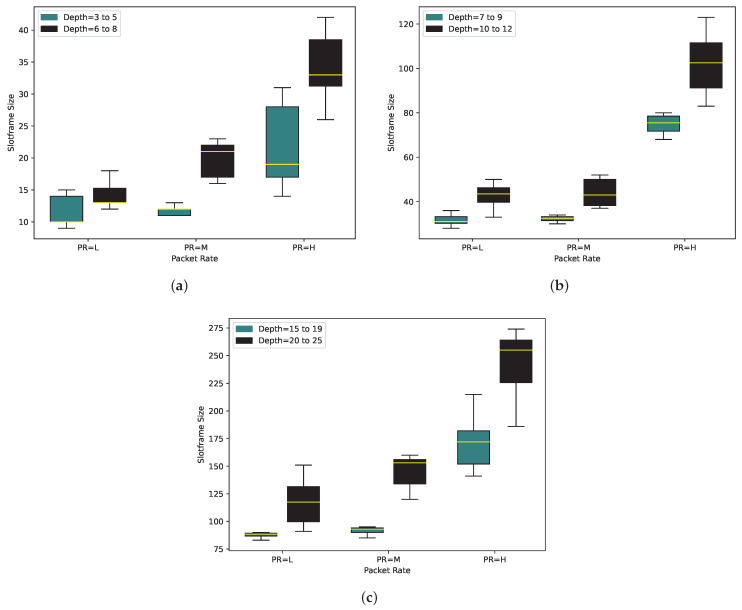
Slotframe size for different tree depths and packet rates in networks with (**a**) 10, (**b**) 20, and (**c**) 50 Nodes.

This experiment aimed to determine the minimum slotframe size required for the TSCH schedule to accommodate all the necessary transmissions in the three previously mentioned approaches. As depicted in Figure 13, it is evident that the slotframe size experiences a significant increase with a higher tree depth and packet rate values. When the tree depth increases, the number of packets that need to be relayed to the root node also rises, resulting in a greater number of EP values for the respective nodes. Consequently, this necessitates the assignment of more transmissions in the TSCH schedule.

Similarly, the packet rate plays a crucial role in determining the slotframe size. Much like the tree depth, an increase in the packet rate leads to a higher EP value, ultimately requiring a larger slotframe size to accommodate all the necessary transmissions. For the 10-node scenario shown in Figure 13a, the minimum slotframe size for packet rates L, M, and H is 9, 11, and 14 for the lower depth values, respectively. However, for the same scenario, these values increase by 33%, 45%, and 85% when the depth increases. It is important to note that the percentage increase is more pronounced when both the packet rate and depth increase simultaneously. It can be observed in Figure 13b,c that the slotframe size increases by increments in the number of nodes too.

**Figure 14:**
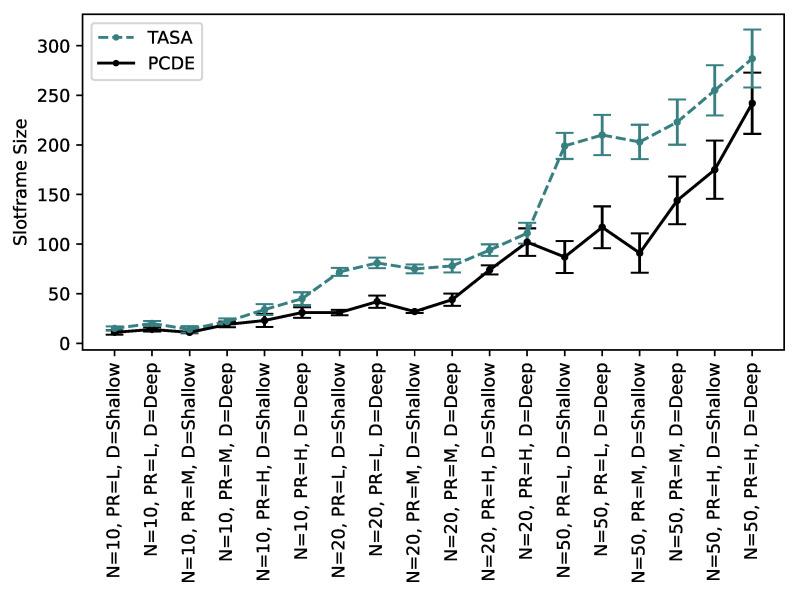
Slotframe size comparison between PCDE and TASA.

Figure 14 is shown to analyze the slotframe size difference more clearly. To quantify the difference between the slotframe sizes of two approaches (PCDE and TASA), we employed the mean absolute difference. This metric is computed by determining the absolute value of the differences between corresponding data points in the two sets. The mean absolute difference in the slotframe size, calculated for 10, 20, and 50 nodes, is 6.8, 31, and 59, respectively. This implies that the slotframe size of TASA closely aligns with PCDE for 10 nodes; however, as the number of nodes increases to 20 and 50, this difference is more notable.

In TASA, nodes generate a constant integer number of packets, and for high packet rates and larger networks, a greater number of slots is required to manage the increased traffic. In PCDE, the objective is to include as many transmissions as possible in the schedule, giving higher priority to nodes with elevated packet rates or a greater number of remaining packets. On the other hand, TASA operates under the assumption that all packets are available at the start of the slotframe. However, through the utilization of TSCH-SIM to simulate real network scenarios, we observed that packets are generated after a warmup period at random intervals based on the specified packet rate. Taking this into consideration, PCDE allocates additional scheduled cells for transmissions, and if a node can transmit during these additional cells, it can achieve the required throughput within a reduced slotframe size.

## 7. Discussion

We demonstrated the PCDE’s performance in terms of various metrics for networks with 10, 20, and 50 nodes. However, as we increased the number of nodes, we observed a sublinear growth in the algorithm’s time complexity. When running the algorithm for networks with over 100 nodes, particularly with medium and high packet rates, it took a day or more to discover a schedule with a minimal slotframe size capable of achieving the required throughput. Nevertheless, this duration can be significantly reduced by upgrading to a faster CPU or adding more RAM.

Considering the potential for network changes in terms of topology and link quality, the algorithm should possess the capability to regenerate the schedule as needed. Given that in the proposed methodology, the optimization objective in each iteration is to minimize delay and generate a new schedule, the resulting schedule can be viewed as a suboptimal solution. However, it might not encompass all the necessary transmissions. Consequently, if a suboptimal schedule is applied to larger networks, there is an expectation of packet loss due to queue overflow, as not all transmissions are scheduled at that point. This emphasizes the challenge of achieving an optimal solution for larger networks while balancing the trade-off between throughput and computational complexity.

## 8. Conclusions

In this paper, we proposed a novel slotframe-length-optimization approach using a Priority-based Customized DE optimization algorithm that considers possible packet collisions and interference. It also supports different packet-generation rates. The presented method finds a schedule with a minimum slotframe length, which will minimize the average delay in the network. A performance analysis using the TSCH-SIM simulator confirmed that the PCDE-optimized schedule outperforms the TASA schedule both in end-to-end delay as well as throughput and *PDR*.

In future work, we are planning to include an adaptive component to the scheduler that can react to changes in the routes of the network as the current static schedule is not ideal as it assumes a static route. We also plan to perform more testing by using a variety of different network topologies as well as determine how sensitive the optimized schedules are to network variations. It is also worth investigating how much further the schedule can be optimized since the current approach results in an overscheduled solution, which can result in scheduled cells that are not being utilized.

## Figures and Tables

**Table 1:** Scenario specifications for performance evaluation.

Scenario	Number of Nodes	Depth	AVG_*NBR*_	Packet Rate
Scenario 1	10	3 to 5	3	L
Scenario 2	10	6 to 8	2	L
Scenario 3	10	3 to 5	3	M
Scenario 4	10	6 to 8	2	M
Scenario 5	10	3 to 5	3	H
Scenario 6	10	6 to 8	2	H
Scenario 7	20	7 to 9	8	L
Scenario 8	20	10 to 12	6	L
Scenario 9	20	7 to 9	8	M
Scenario 10	20	10 to 12	6	M
Scenario 11	20	7 to 9	8	H
Scenario 12	20	10 to 12	6	H
Scenario 13	50	15 to 19	15	L
Scenario 14	50	20 to 25	9	L
Scenario 15	50	15 to 19	15	M
Scenario 16	50	20 to 25	9	M
Scenario 17	50	15 to 19	15	H
Scenario 18	50	20 to 25	9	H

**Table 2:** Evaluation parameters and their specified ranges.

Parameter	Description	Range
A	Target Area	200 ∗ 200 m^2^
*N*	Total number of nodes	10, 20, 50
$S_{i}$	Sensor with ID *i*	i∈[1…N]
$AVG_{NBR}$	Average number of neighbors for each node	Varies by topology and N value
PR	Packet rate	L, M, H
*R*	Sensing range of node	40 m
$n_{ch}$	Number of channel offset	4
*D*	Depth of tree	Varies by topology and N value
LI	Maximum number of Local Iterations	20, 50

**Table 3:** Simulation parameters used in TSCH-SIM simulation.

Parameter	Value
SIMULATION_DURATION	3000 s
APP_WARMUP_PERIOD_SECOND	1500 s
LINK_MODEL	Logistic Loss
APP_PACKET_SIZE	100
MAC_MAX_RETRIES	7
MAC_QUEUE_SIZE	20
LOGISTICLOSS_TRANSMIT_RANGE_M	40 m
TSCH_SCHEDULE_DEFAULT_LENGTH	Derived slotframe size
ROUTING_ALGORITHM	ManualRouting
SCHEDULING_ALGORITHM	ManualScheduler
TIME_SLOT_DURATION	10 ms

**Table 4:** Performance results for different network scenarios for the PCDE algorithm (LI = 20).

Scenario	Delay (ms)	PDR	Throughput	Time	Duty Cycle	Slotframe Size
Scenario 1	0.23	100%	1.1	401	100%	11
Scenario 2	0.43	100%	1.17	544	100%	14
Scenario 3	0.22	100%	1.6	399	100%	11
Scenario 4	0.7	100%	1.48	1122	100%	19
Scenario 5	1.23	100%	8.37	1629	100%	23
Scenario 6	1.14	100%	9.34	2902	100%	31
Scenario 7	1.44	100%	2.48	1969	100%	32
Scenario 8	1.4	98%	2.59	3647	100%	43
Scenario 9	1.42	99.8%	9.03	2040	100%	32
Scenario 10	2.08	98.6%	5.74	4154	100%	44
Scenario 11	2.7	98.4%	11.43	9903	100%	75
Scenario 12	3	98.6%	11.5	15,980	100%	105
Scenario 13	0.2	98.9%	0.7	10,225	100%	88
Scenario 14	0.23	96%	0.89	13,769	100%	118
Scenario 15	0.3	98.7%	6.66	10,680	100%	92
Scenario 16	0.45	94.3%	1.35	16,897	100%	144
Scenario 17	1.2	96.2%	2.67	20,554	100%	176
Scenario 18	1.5	94%	3.5	28,299	100%	244

**Table 5:** Performance results for different network scenarios for TASA.

Scenario	Delay (ms)	PDR	Throughput	Duty Cycle	Slotframe Size
Scenario 1	3	99.5%	0.9	100%	15
Scenario 2	4.9	99.1%	0.88	100%	20
Scenario 3	3.5	99.6%	1.5	100%	14
Scenario 4	4.4	99.3%	1.38	100%	22
Scenario 5	4.5	97%	7.6	100%	34
Scenario 6	5	97.6%	7.01	100%	45
Scenario 7	8	98.1%	1.5	100%	72
Scenario 8	8.8	97%	1.8	100%	81
Scenario 9	7.4	97.8%	5.9	100%	75
Scenario 10	9.6	96%	4.8	100%	78
Scenario 11	11	95.1%	8.87	100%	94
Scenario 12	12.3	95.5%	8.4	100%	111
Scenario 13	11.1	93.7%	1.9	100%	199
Scenario 14	12.6	94%	1.6	100%	210
Scenario 15	11.9	94.5%	6.66	100%	203
Scenario 16	14.87	94.7%	1.35	100%	223
Scenario 17	17.2	90.1%	2.67	100%	255
Scenario 18	21	88.5%	3.5	100%	287

## Data Availability

Data are contained within the article.

## Abbreviations

The following abbreviations are used in this manuscript:

AMUS	Adaptive Multi-hop Scheduling
CDE	Customized Differential Evolution
CMAB	Combinatorial Multiarmed Bandit
DE	Differential Evolution
EP	Expected packet
FDMA	Frequency Division Multiple Access
LI	Local Iteration
LLR	Linear Learning Rewards
MAC	Medium Access Control
MST	Minimum Spanning Tree
OSCAR	Optimized Scheduling Cell Allocation Algorithm
PCDE	Priority-based Customized Differential Evolution
PDR	Packet Delivery Ratio
PR	Packet rate
TASA	Traffic-Aware Scheduling Algorithm
TDMA	Time Division Multiple Access
TSCH	Time-Slotted Channel Hopping
